# A Mobile Phone–Based Telemonitoring Program for Heart Failure Patients After an Incidence of Acute Decompensation (Medly-AID): Protocol for a Randomized Controlled Trial

**DOI:** 10.2196/15753

**Published:** 2020-01-22

**Authors:** Emily Seto, Heather Ross, Alana Tibbles, Steven Wong, Patrick Ware, Edward Etchells, Jeremy Kobulnik, Tamanna Chibber, Stephanie Poon

**Affiliations:** 1 Institute of Health Policy, Management and Evaluation University of Toronto Toronto, ON Canada; 2 Centre for Global eHealth Innovation Techna Institute University Health Network Toronto, ON Canada; 3 Ted Rogers Centre for Heart Research University Health Network Toronto, ON Canada; 4 Department of Medicine University of Toronto Toronto, ON Canada; 5 Peter Munk Cardiac Centre University Health Network Toronto, ON Canada; 6 Faculty of Medicine University of British Columbia Vancouver, BC Canada; 7 Department of Medicine Sunnybrook Health Sciences Centre Toronto, ON Canada; 8 Centre for Quality Improvement and Patient Safety University of Toronto Toronto, ON Canada; 9 Department of Cardiology University Health Network Toronto, ON Canada; 10 Department of Cardiology Mount Sinai Hospital Toronto, ON Canada; 11 Faculty of Medicine University of Calgary Calgary, AB Canada

**Keywords:** heart failure, telemedicine, mobile phone, patient monitoring, randomized controlled trial

## Abstract

**Background:**

Patients with heart failure (HF) are at the highest risk for hospital readmissions during the first few weeks after discharge when patients are transitioning from hospital to home. Telemonitoring (TM) for HF management has been found to reduce mortality risk and hospital readmissions if implemented appropriately; however, the impact of TM targeted for patients recently discharged from hospital, for whom TM might have the biggest benefit, is still unknown. Medly, a mobile phone–based TM system that is currently being used as a standard of care for HF at a large Canadian hospital, may be an effective tool for the management of HF in patients recently discharged from hospital.

**Objective:**

The objective of the *Medly-After an Incidence of acute Decompensation* (Medly-AID) trial is to determine the effect of Medly on the self-care and quality of life of patients with HF who have been recently discharged from hospital after an HF-related decompensation.

**Methods:**

A multisite multimethod randomized controlled trial (RCT) will be conducted at 2 academic hospitals and at least one community hospital to evaluate the impact of Medly-enabled HF management on the outcomes of patients with HF who had been hospitalized for HF-related decompensation and discharged during the 2 weeks before recruitment. The trial will include 144 participants with HF (74 in each control and intervention groups). Control patients will receive standard of care, whereas patients in the intervention group will receive standard of care and Medly. Specifically, patients in the intervention group will record daily weight, blood pressure, and heart rate and answer symptom-related questions via the Medly app. Medly will generate automated patient self-care messages such as to adjust diuretic medications, based on the rules-based algorithm personalized to the individual patient, and send real-time alerts to their health care providers as necessary. All patients will be followed for 3 months. Primary outcome measures are self-care and quality of life as measured through the validated questionnaires Self-Care of Heart Failure Index, EQ-5D-5L, and the Kansas City Cardiomyopathy Questionnaire-12. Secondary outcome measures for this study include cost of health care services used and health outcomes.

**Results:**

Patient recruitment began in November 2018 at the Sunnybrook Health Sciences Centre, with a total of 35 participants recruited by July 30, 2019 (17 in the intervention group and 18 in the control group). The final analysis is expected to occur in the fall of 2020.

**Conclusions:**

This RCT will be the first to assess the effectiveness of the Medly TM system for use following discharge from hospital after a HF-related decompensation.

**Trial Registration:**

ClinicalTrials.gov NCT03358303; https://clinicaltrials.gov/ct2/show/NCT03358303

**International Registered Report Identifier (IRRID):**

DERR1-10.2196/15753

## Introduction

### Background

Over 670,000 Canadians live with a diagnosis of heart failure (HF) [1,2] and the prevalence is increasing [3]. HF is associated with a high mortality rate of 30% at 1 year [4] and high health care costs largely attributed to repeated hospital admissions [3]. The highest risk for rehospitalization is during the first few weeks after discharge when patients are transitioning from hospital to home [5]. Among all patient groups, patients with HF have the highest readmission rate within 30 days, at approximately 20% [5,6]. The 2019 Report on the Health of Canadians by the Heart and Stroke Foundation found that HF costs Can $2.8 billion per year and is the third most common reason for hospitalization (after respiratory disease and heart attack, which are both associated with HF) [1].

Up to 40% of readmissions have been found to be preventable [7] and related to suboptimal transitional care because of the lack of coordination and continuity of care as patients transfer between health care settings and providers [8]. To address the issue of frequent readmissions, quality improvement initiatives have included increasing education during vulnerable transition periods, such as just before discharge; rapid 7-day clinical reassessment; and improved discharge summaries [9-12]. Telemonitoring (TM) has also emerged as a potential approach to improve HF management during the transition from hospital to home, by enabling providers to closely monitor relevant physiological parameters and symptoms in real time.

Recent systematic reviews have found that TM for HF management reduces mortality risk and hospital readmissions, and more frequent transmission of patient data increases its effectiveness [13,14]. A large-scale randomized controlled trial (RCT) by Koehler et al [15] found reductions in all-cause mortality and length of hospital admissions. However, 3 previous large-scale trials have failed to show the benefits of TM [16-18]. This inconsistency in the findings of HF TM can be attributed to the heterogeneity of the trials with variations in the characteristics of the interventions, patients, providers, organizations, and structural characteristics (eg, policies and incentives). A review by Ware et al hypothesized that inconsistencies among the results of different TM studies may also be largely because of the lack of tailoring the TM program to the population and context for successful implementation [19].

Medly is a highly automated and user-centered mobile phone–based TM system developed at the University Health Network, Toronto, Canada. Medly has been evaluated through an RCT with outpatients with HF at the Ted Rogers and Family Centre of Excellence in Heart Function, University Health Network, a tertiary academic hospital. The RCT found improvements in quality of life and self-care management, a reduction in brain natriuretic peptide (BNP) levels, and an increase in left ventricular ejection fraction over a 6-month period [20,21].

### Objectives

The main objective of the *Medly-After an Incidence of acute Decompensation* (Medly-AID) trial is to determine the effect of Medly on the self-care and quality of life (primary outcome measures) of patients with HF who have been recently discharged from hospital after a HF-related acute decompensation. Acute decompensation is when there is a sudden worsening of signs and symptoms of HF, which often leads to a visit to the emergency department (ED) or hospitalization. Secondary outcome measures include the cost of health care services used and health outcomes. A main difference between the Medly-AID trial and the previous Medly RCT is that the Medly-AID trial will be restricted to patients who have been discharged from hospital during the 2 weeks before recruitment, thus targeting the patients at the highest risk for rehospitalization.

## Methods

### Study Overview

Medly-AID is a multisite multimethod RCT with 144 patients with HF. Patients are enrolled in the study for 3 months. The Medly TM system will be implemented at 2 academic hospitals, namely, Sunnybrook Health Sciences Centre and Mount Sinai Hospital, both in Toronto, Canada. A third site, a community hospital, will also be included in the trial. This study has received approval from the research ethics boards at the Sunnybrook Health Sciences Centre (research ethics board number 143-2017) and the University Health Network (research ethics board number 17-5887), where the patient data will be stored, and the submission to Mount Sinai Hospital is in review. The trial has also been registered at ClinicalTrials.gov (NCT03358303) since November 30, 2017.

### Sample Size Calculation

A sample size calculation was based on the Self-Care of Heart Failure Index (SCHFI), using a population standard deviation of 20 and an effect size of 10 as determined in previous studies (alpha=.05; power=0.8) [22]. The required sample size was calculated to be 63 per group. To compensate for patients who will be lost to follow-up during the course of the study, 72 patients will be recruited for the intervention group and 72 patients for the control group.

### Medly Telemonitoring System

**Figure 1:**
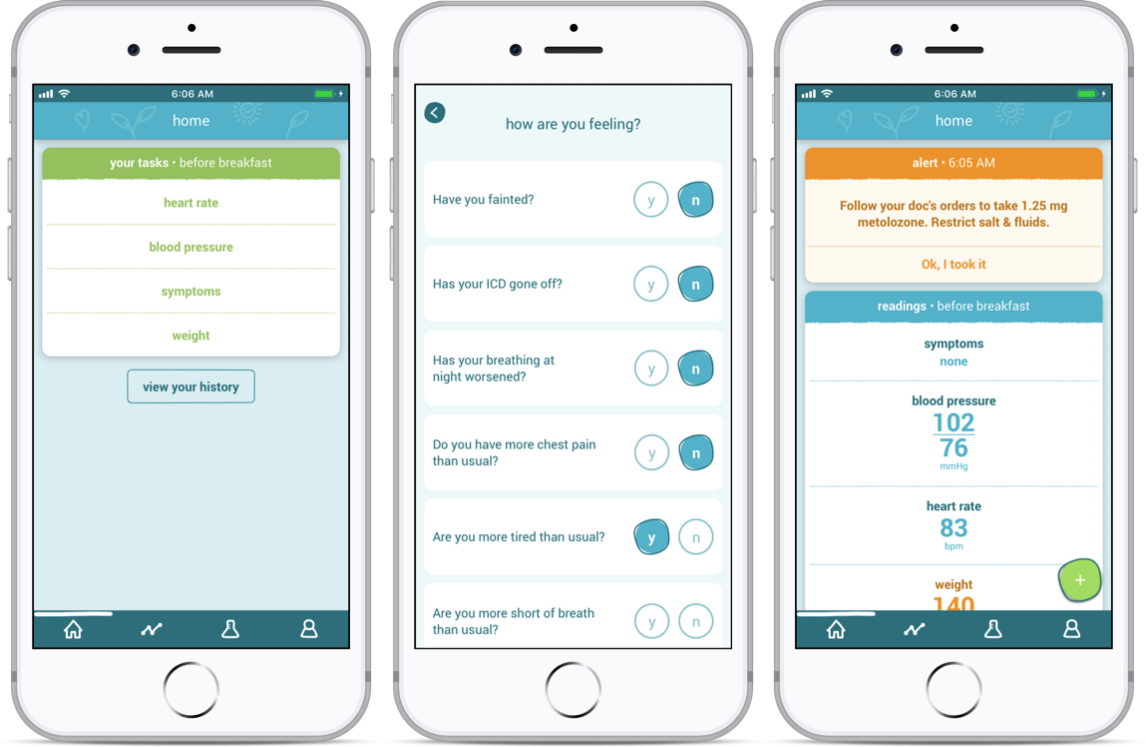
Sample screenshots of the Medly app.

The mobile phone–based TM system, Medly, enables patients with HF to monitor their weight (A&D Medical Bluetooth weighing scales), blood pressure, and heart rate (A&D Medical Bluetooth blood pressure monitors) using wireless home medical devices. Medly was developed using a user-centered design approach that included iterative rounds of usability testing. See Figure 1 for screenshots of the Medly app. Patients are instructed to measure their weight and blood pressure (systolic and diastolic values) with the provided medical devices and to answer simple questions regarding their symptoms first thing each morning after the patient relieves themselves in the washroom and before eating or drinking. Heart rate values are also recorded simultaneously as they are taking their blood pressure values. Automated reminder phone calls are sent to the patient’s own phone if they do not take their measurements by 10 am, but patients can opt out of this feature. If the patient is exhibiting HF-related symptoms such as increased shortness of breath or chest pain during the rest of the day, they are instructed to measure their blood pressure and to record their symptoms through the Medly app. The measurements are automatically and wirelessly transmitted via Bluetooth to the mobile phone with the Medly app and then relayed to a secure data server. On the basis of their readings and reported symptoms, automated and individualized self-care messages, such as to adjust diuretic medication and restrict fluid and sodium consumption, are sent to the patients [20]. If there are signs of clinical deterioration, an alert is sent to the clinician responsible for the care of the patient. Clinicians can access all relevant patient information through a secure Web portal, which allows them to access trends and historical data for each individual. These data enable clinicians to identify health deteriorations early and to address any issues remotely (eg, providing further self-care guidance or altering the patient’s care plan in such a way that stabilizes the patient and ideally avoids a rehospitalization). The patient information on the mobile phone provided to patients can be remotely deleted if the phone is reported stolen or lost.

### Study Protocol

#### Patient Recruitment and Randomization

Patients are recruited within approximately 2 weeks post discharge, often during their regularly scheduled clinician visits for postdischarge care at the participating sites. After clinicians determine if the patient is eligible (inclusion and exclusion criteria can be found in Textboxes 1 and 2, respectively), the patient is asked if they are willing to speak to the study coordinator about enrollment. Informed written consent is obtained from patients by the research coordinator. Each patient is randomized to either the TM intervention or standard care control group. Block randomization is used with random block sizes of 2, 4, or 6 for each site. A Web-based computer-generated randomization tool, Sealed Envelope (Sealed Envelope Ltd.) [23], is used to determine the randomization order. Sequentially numbered envelopes are used to determine if the patient is in the intervention or control arm. The envelopes are prepared by a researcher outside of the study team. The study coordinator and patient are blinded to which group the patient is assigned until the patient has consented to participate in the study. Each participant in the control and intervention group receives a Can $25 Shoppers Drug Mart gift card to compensate them for their time in participating in the study.

**Patient inclusion criteria.**

- Adults (18 years or older)
- Hospitalization duration for decompensated heart failure >24 hours
- Patient speaks and reads English adequately to provide informed consent and understand the text prompts in the app (or has an informal caregiver who can translate the prompts for them)
- Ability to comply with using Medly (eg, able to stand on the weight scale and able to answer symptom questions)

**Patient exclusion criteria.**

- Dementia or uncontrolled psychiatric illness
- Patients who will require inpatient rehabilitation after discharge
- Participating in another clinical trial that may confound the results
- Residents of long-term care facilities
- Terminal diagnosis of any health condition with a life expectancy of <1 year
- Unable to provide informed consent
- Unable to speak or read English

#### Intervention Versus Control Groups

Patients in the intervention group will receive standard of care and also Medly, whereas the patients in the control group will receive standard of care. Standard of care will comprise the usual follow-up and routine management performed by each participating institution for patients with HF. Patients in the intervention group will receive the Medly kit to take home after being trained on how to use Medly. The Medly kit includes a mobile phone, with the Medly app, provided to the patient as well as a Bluetooth-enabled weighing scale and blood pressure monitor. Each patient will be followed for 3 months post discharge.

#### Study Outcome Measures

The primary outcome measures will be self-care as measured through the SCHFI [22] and quality of life as measured through the Kansas City Cardiomyopathy Questionnaire-12 [24] and the EQ-5D-5L [25]. Additional measures will include compliance with Medly utilization determined through the data on the Medly-AID servers regarding the frequency that patients took measurements; shortness of breath as measured using a visual analog scale for dyspnea in the patient questionnaires; and clinical status as measured by the New York Heart Association class, left ventricular ejection fraction, and BNP/N-terminal proBNP (NT-proBNP) levels (obtained via the electronic medical records). Health service utilization will be assessed by 30-day HF readmission rates, HF length of stay, and number of visits to the ED. The cost of the intervention will be determined by tracking the equipment costs and staff resources for clinical, technical, and administrative support and will be compared with the cost of health services. Creatinine, sodium, and potassium levels will also be assessed through routine blood tests from the patients, usually conducted at the participating institution’s laboratories. These values will be obtained through the patients’ electronic medical records. The schedule for data acquisition is depicted in Table 1.

**Table 1:** Schedule for data acquisition indicated by checkmarks at the specified time point.

Data Collected			Baseline		2 weeks		1 month		3 months
**Questionnaires**									
	Demographics	✓		—^a^		—		—	
	EQ-5D-5L	✓		—		—		✓	
	Self-Care of Heart Failure Index	✓		—		—		✓	
	Kansas City Cardiomyopathy Questionnaire-12	✓		—		—		✓	
	Visual analog scale for dyspnea	✓		—		—		✓	
	Perceptions of Medly program (intervention group only)	—		—		—		✓	
**Health service utilization**									
	Number of hospitalizations in the previous 3 months	✓		—		—		✓	
	Number of days in the hospital in the previous 3 months	✓		—		—		✓	
	Number of emergency department visits in the previous 3 months	✓		—		—		✓	
	Number of clinic visits in the previous 3 months	✓		—		—		✓	
	Number of readmissions to hospital since recruitment	—		—		✓		—	
**Blood test outcomes**									
	N-terminal proBNP^b^/BNP levels	✓		✓		✓		✓	
	Creatinine	✓		✓		✓		✓	
	Sodium	✓		✓		✓		✓	
	Potassium	✓		✓		✓		✓	

^a^Not applicable.

^b^BNP: brain natriuretic peptide.

### Data Collection

Prestudy and poststudy questionnaires will comprise the aforementioned validated survey tools, whereas the poststudy questionnaire for the patients in the intervention group will have additional questions related to their perceptions of Medly. Prestudy and poststudy questionnaires will be administered to both control and intervention groups, which may be completed in the clinic or at home. Participants choosing to complete the questionnaires at home will be provided with prestamped envelopes addressed to the research team. The prestudy questionnaire will be provided to the participants at the time of enrollment, whereas the poststudy questionnaire will be provided after a follow-up appointment with the participant’s clinician at the time of study completion (3 months after enrollment). Data on the 30-day HF readmission rates; HF length of stay; number of visits to the ED; and clinical measures, including creatinine, sodium, potassium, left ventricular ejection fraction, and BNP/NT-proBNP levels, will be determined through the hospital’s electronic medical records and manual chart reviews.

#### Semistructured Interviews

Using purposive sampling, semistructured interviews at the end of the study will also be conducted with a subset of patients in the intervention group to receive feedback from patients with different experiences using Medly-AID. Participants for the interviews will be selected based on varying adherence to taking measurements, number of readmissions to hospital, difficulties in using Medly, etc. Interviews will be conducted until data saturation is reached (ie, interviewer determines no new relevant information is being collected), which is typically 15 to 20 participants. The interviews are expected to last for 20 min to 30 min and will be conducted in a quiet and private space within the clinic (eg, consultation room) during a regular clinic visit or over the telephone. All interviews will be audiotaped and transcribed for later analysis. Clinicians providing care for patients using Medly-AID will also undergo a semistructured interview post study to determine their perceptions of the use and impact of Medly-AID. It is anticipated that the clinician interviews will last for 15 min to 45 min.

#### Data Analysis

For each outcome measure, normality of the data will be verified using the Kolmogorov-Smirnov and Shapiro-Wilk tests of normality. Between-group analyses using independent Student *t* tests and Mann-Whitney tests (for normally and nonnormally distributed data, respectively) will be performed. Paired Student *t* tests and Wilcoxon signed rank tests will also be performed to compare baseline and poststudy data for both the intervention and control groups as appropriate. The statistical analyses will be performed using the statistical software SPSS 17.0 (IBM Corporation). Statistical significance will be considered at *P*<.05, unless otherwise specified. All reported test results will be 2-tailed.

The transcribed interview data will be analyzed using conventional content analysis, whereby 2 researchers will analyze the transcripts for themes independently [26]. The 2 researchers will then discuss the themes and issues that emerge until a consensus is reached. Triangulation, which is validation of the findings through the collection of data from different sources or methods, will be used for this study. Specifically, the interview findings will be used to try to confirm and explain the results of the quantitative data, including health services utilization and Medly usage.

## Results

Patient recruitment began in November 2018 in the Sunnybrook Health Sciences Centre. The study is currently ongoing, with a total of 35 participants (17 in the intervention group and 18 in the control group) as of July 30, 2019. The anticipated date for final analysis of the data is fall of 2020.

## Discussion

### Strengths and Limitations of the Study

This RCT aims to investigate the impact of a mobile phone–based HF TM system on patients with HF recently discharged from hospital for decompensated HF. Previous studies on HF TM have yielded contradictory findings, with some showing benefits to patient outcomes, including reductions in hospitalizations, whereas other trials have found no benefits [16-18]. Factors influencing the benefits include the features of the TM system, patient population, study design, and the implementation plan [27,28]. A study by Ware et al [29] summarizes the available evidence on HF TM effectiveness and discusses the complexity in accounting for these factors. This was followed by a literature review and white paper from the Heart Failure Society of America, which concluded that HF remote monitoring was most beneficial for patients at most risk for decompensation and hospitalization, suggesting that null findings from previous studies might be attributed to including patient participants with HF who were already stable [30]. In this trial, we are employing a TM system, Medly, that is currently being used as a standard of care in an outpatient heart function clinic at a large academic hospital, with proven beneficial features in a mixed population of stable and unstable patients [20,21]. Therefore, this study is intended to test the impact of the same system with a target population that is hypothesized to benefit the most (ie, patients who have been discharged from hospital and are at the highest risk for readmissions).

In terms of the study design, a multimethod approach will be used, which will enable triangulation of data from questionnaires, patient data from home monitoring, Medly usage data, patient medical records, costing information, and interviews. This will allow for a comprehensive understanding of the impact of Medly on the end users, clinical workflows, cost savings, and perceptions of TM by clinicians and patients. For example, the interview data may help to understand the features of Medly that are useful and those that are not useful, as well as to understand how patients with HF recently discharged from hospital might differ in their perceptions and adherence to TM compared with other patients with HF. Finally, the extensive expertise from careful TM program implementation planning will be leveraged for the planning of this RCT [19,27,31].

A limitation of this study includes the relatively small patient sample size. It is expected that this trial will be underpowered to detect the impact of Medly on hospital readmission rates. Benefits of Medly on the primary outcomes would provide evidence to support a future larger clinical trial to determine the effectiveness of Medly on hospital readmission rates. The intent of the study is to deploy Medly within representative academic and community hospitals within Ontario. However, the number of deployment sites will be relatively small (3 to 4 sites), which is another limitation as it may impact the generalizability of the findings.

### Significance of the Research

The rising incidence of HF [32,33] highlights the importance of developing and validating novel technologies or management strategies. This trial will be one of the few studies that will provide evidence of the impact of HF TM specifically for patients recently discharged from hospital. The trial will investigate the impact through various lenses, including patient self-care and quality of life, clinician experience and impact to their workflow, costs to the health care system, and patient health outcomes. It is anticipated that this study will help inform how to optimize the future scale and spread of TM to support patients and clinicians in the management of HF.

## Appendix

Multimedia Appendix 1 Peer-reviewer report from the Sunnybrook Alternative Funding Plan.

## Abbreviations

BNP: brain natriuretic peptide
ED: emergency department
HF: heart failure
Medly-AID: Medly-After an Incidence of acute Decompensation
NT-proBNP: N-terminal probrain natriuretic peptide
RCT: randomized controlled trial
SCHFI: Self-Care of Heart Failure Index
TM: telemonitoring
