# Supplementary material for: A Mobile Phone–Based Telemonitoring Program for Heart Failure Patients After an Incidence of Acute Decompensation (Medly-AID): Protocol for a Randomized Controlled Trial
Source: JMIR Res Protoc. 2020 Jan 22;9(1):e15753. doi: 10.2196/15753 (PMC7003117; doi:10.2196/15753)
Supplement: Multimedia Appendix 1 [file resprot_v9i1e15753_app1.pdf]

Sunnybrook
ALTERNATIVE FUNDING PLAN

Dr. Stephanie Poon
Department of Medicine
Sunnybrook Health Sciences Centre

January 10, 2018

RE: 2017/2018 Innovation Fund

Dear Dr. Poon,

Congratulations! Your proposal to the Sunnybrook AFP Innovation Fund review panel titled, *"Medly-AID": Effect of Medly, a mobile phone-based telemonitoring program on the outcome of heart failure patients After an Incidence of acute Decompensation,* has been chosen to be shortlisted for submission to the Innovation Fund Provincial Oversight Committee (IFPOC).

This is not yet confirmation of an award, although in the past all projects we have submitted have been funded. Final results will be communicated to us by early March and funding will arrive in mid-April. In order for your project to be ready to go at this time we recommend you begin the REB process and that you obtain any cross-hospital agreements required for your project. These administrative tasks can delay the start of a project significantly.

This was a competitive year, with eleven of the forty five original LOI submissions eventually chosen to be recommended for funding to the Innovation Funding Provincial Oversight Committee.

The review panel has provided the following feedback on your proposal for you to consider as you implement your project. Some may strengthen the project and its ultimate impact:

- Unique initiative that combines the use of a mobile phone-based telemonitoring system (Medly), with personalized automated instructions for each patient on how to adjust their diuretics based on daily physiologic parameters and symptoms, as well as built in alerts for their health care providers.
- Has the potential of shifting care to an ambulatory manner and giving patients a degree of autonomy.
- Strong team. Capable of doing project. Has included site investigators as collaborators; feasible with 3 centres.
- Clearly stated hypothesis that is in line with the background provided. Objectives as well are clear.
- This device and strategy for home management is innovative and has high potential to be used for this population and could be transferred to other medical fields.
- Strong team with experience in Heart failure and use of this device. Also methodological expertise.
- Major public health problem. Intervention/innovation has potential for major impact and to be a precedent for similar innovations in other medical disciplines.

- A clearly stated research question would be preferable. Many outcomes are described. Primary outcome is presumably the SCHFI since it was used in the sample size calculation. Justification of this as the best primary outcome would be an improvement to the proposal.
- Having qualitative portion to assess patient and physician experience with the device is a strength of the proposal.
- Multiple outcomes are planned to be analyzed. Multiple comparisons will result and a clearly identified research question with the most important primary outcome identified would be a better trial design. Multiple measurements of both outcome and safety measures is a strength.
- A multicenter RCT with heart failure patients assigned to either usual care or to post discharge management with a Medly smartphone app/monitoring device. Hypothesis of improved heart failure management with this device/regimen. High potential for improved care based on an innovative use of technology. Technology has been already created and piloted. A good plan to evaluate a device with potential before widespread adoption.
- Study is about to be up and running based on other funding. The budget lays out how the AFP funds will be leveraged to complete this project.
- Building on a previous project. Project is multi-site and also involving a community hospital which allows for more generalizability. Clearly stated objectives with clear metrics to be used to determine outcome measure. Methodology is that of an RCT where patients who meet inclusion/exclusion criteria will then randomized to telemonitoring or standard approach in care. Random block sizes will be used. Is centre included as a stratification variable for the treatment allocation to be somewhat balance at all sites? Protocol is well-defined and has been utilized in a previous trial.
- Because Medly can be viewed as a device, has this device been approved for the use being considered for the project. In other words, will it need Health Canada approval or just clearance?
- Demonstrated need for this. It is an extension of an already established intervention to determine generalizability? Providing the phones is a strength as I imagine some older patients may not have a phone/data plan. How do you define "compliance with Medly utilization"?

The following reader remarks were not considered in your ranking of application:

- As the project is mobile phone based by definition, there is a significant technology component to this project. Budget calls for research analyst programmer and technical support budget lines again critical to IT projects. Therefore proposal requires sign off by CCO as per IFPOC document.
- Also, study specifically indicates this is a multisite study and dependent in part on funding from elsewhere. Have similar AFP grants been submitted to JDMI AFP or St. Mikes hospital? This has not been adequately noted in the appropriate check boxes. More details about Medley is required.
- Is this a third party vendor that stands to gain commercially from results of research conducted here? I note devices are circulated freely. What is the relationship of PI or institutions involved to Medley etc.?
- If intervention is at patient level, how is success with the most severely affected patients not transferable to less severely affected patients (with better LVEF) if the objectives are to test feasibility, acceptability and improved self-care? One can certainly make a case for the third objective separately which may be more relevant given the prior study did not find a difference between control and Medley group who did not attend the clinic other than quality of life scores.
- Given that references to methodology repeatedly refer to prior papers and “multiple studies” previously studying the technology (mentioned under team section), it is unclear what the novelty/innovation of the present proposal is. What lessons were learned from that study to justify another? How many patients based on prior data are needed to show benefit?

Thank you for your submission and we hope you can utilize this feedback to enhance your research.

Dr. Rajin Mehta
Chair
Sunnybrook AFP Board of Directors

Dr. Richard Aviv
Chair
Sunnybrook Innovation Fund Sub-Committee
